# Dynamic endoscopic progression of gastrointestinal tract involvement in Langerhans cell histiocytosis: A pediatric case report

**DOI:** 10.1002/deo2.70023

**Published:** 2024-09-27

**Authors:** Jianwei Pan, Bo Liu, Huihua Zhang, Zhongyue Li

**Affiliations:** ^1^ Department of Pediatrics, The Fourth Affiliated Hospital of School of Medicine, and International School of Medicine, International Institutes of Medicine Zhejiang University Yiwu China; ^2^ Department of Gastroenterology Children's Hospital of Chongqing Medical University Chongqing China

**Keywords:** dabrafenib, endoscopy, gastrointestinal tract, Langerhans cell histiocytosis, pediatric

## Abstract

Gastrointestinal tract involvement in Langerhans cell histiocytosis (LCH) is extremely rare, with limited documentation of endoscopic manifestations. We report a 19‐month‐old girl who presented with repeated diarrhea and bloody stools, accompanied by recurrent pulmonary infections, anemia, hypoproteinemia, thrombocytopenia, coagulopathy, and hepatosplenomegaly with lymphadenopathy. Initial treatment with antibacterial agents, mesalazine, thalidomide, and prednisone led to temporary improvement; however, the symptoms repeatedly relapsed. She underwent three digestive endoscopies, but until the third endoscopy, a definitive diagnosis of Langerhans cell histiocytosis was established through biopsy. While upper gastrointestinal tract findings were not significant, notable changes were observed in the colorectal region. A colonoscopy revealed progression from erythema to diffuse hyperemia and edema, with erythema, erosion, and superficial ulcers extending into the distal ileal mucosa. Genetic analysis identified a BRAF‐V600E mutation. Following treatment with chemotherapy (vincristine and prednisone) and the BRAF inhibitor dabrafenib, the patient demonstrated significant clinical improvement within days. At the 1‐year follow‐up, the patient had normal bowel movements and a weight gain of 2.5 kg. Early gastrointestinal endoscopy with multiple biopsies in suspected children can facilitate early detection. Dabrafenib is a viable treatment option for Langerhans cell histiocytosis.

## INTRODUCTION

Langerhans cell histiocytosis (LCH) is a clonal, neoplastic proliferative disease characterized by the proliferation of immature dendritic cells (CD1a+/CD207+), leading to tissue and organ infiltration and subsequent functional impairments.^1^ LCH can involve a single or multiple organs or systems, with bones and skin being the most commonly affected sites. Gastrointestinal tract (GIT) involvement is extremely rare and is associated with a poor prognosis.^2^ It can occur at any age but predominantly affects girls under the age of 2. Endoscopic and pathological examinations are essential for diagnosis; however, related endoscopic manifestations are few documented, especially the dynamic endoscopic findings. Herein, we report a pediatric case of GIT‐LCH who underwent three digestive endoscopies and was successfully treated with dabrafenib.

## CASE REPORT

A 19‐month‐old girl was referred to our department with a 10‐month history of recurrent diarrhea and bloody stools. At nearly 9 months of age, she was first hospitalized due to recurrent fevers and diarrhea lasting over 20 days. During this period, she developed severe infections and respiratory distress necessitating ventilatory support. Laboratory findings revealed hypoproteinemia (albumin 21.9 g/L), liver failure, and clinical features resembling hemophagocytic syndrome (HPS), including coagulopathy, severe anemia (hemoglobin 55 g/L), thrombocytopenia (platelet 31×10^9^/L), elevated ferritin (1215 µg/L), reduced fibrinogen (1.06 g/L), and hepatosplenomegaly with lymphadenovarix. Bone marrow examination did not demonstrate hemophagocytosis, and aside from a positive cytomegalovirus test, other infectious workups were negative. Initially, the patient was diagnosed with infectious diarrhea and HPS and was treated with cefoperazone, imipenem, vancomycin, high‐dose methylprednisolone (15 mg/kg), and oral prednisone, leading to an initial improvement in her symptoms. However, her diarrhea and bloody stools recurred, resulting in multiple subsequent hospital admissions.

**FIGURE 1:**
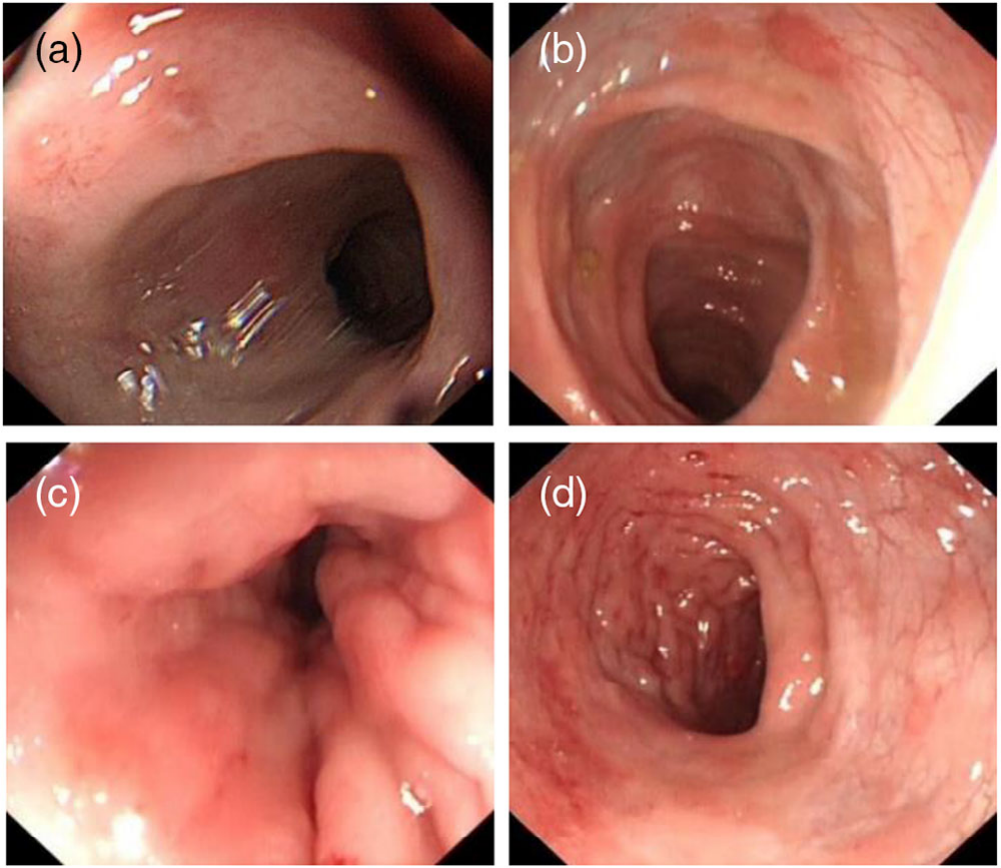
Endoscopic images of the patient at 1 year old. (a) Congestion, edema, and erosion of the duodenal bulb mucosa. (b–d) Congestion and edema of the colorectal mucosa with mucosal erythema.

**FIGURE 2:**
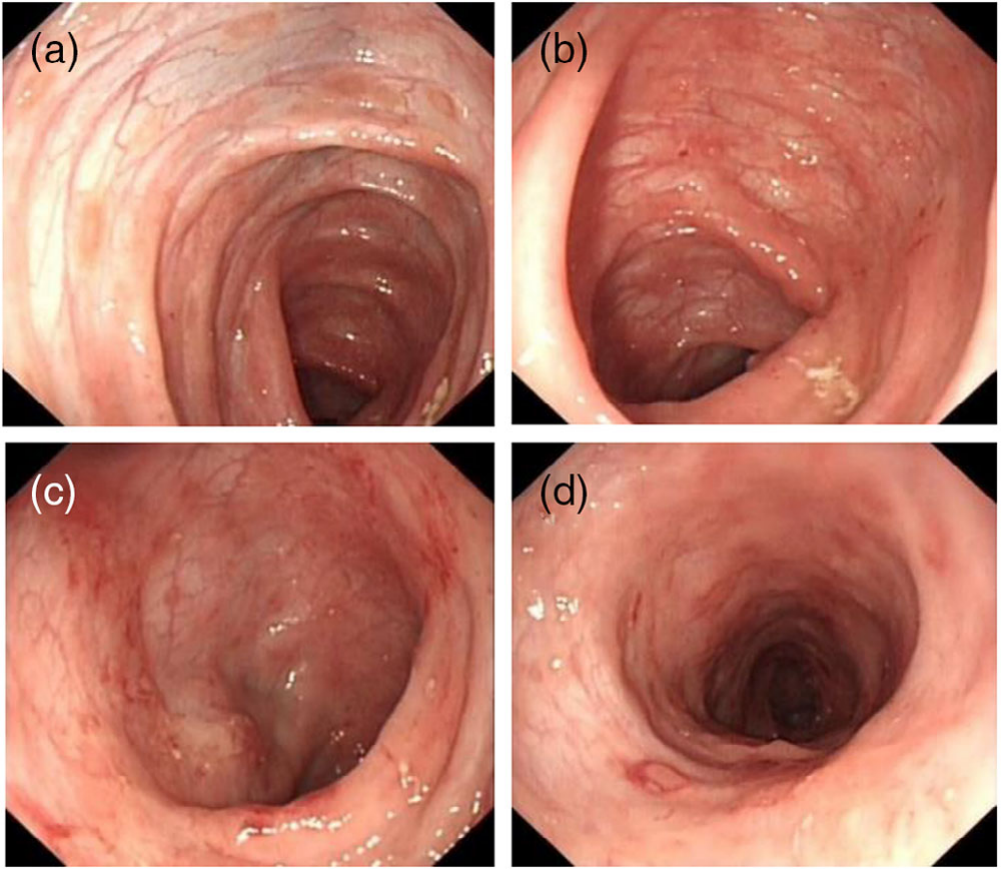
Endoscopic images of the patient at 14 months old. (a–d) Congestion and edema of the colorectal mucosa with erythema and erosion, more densely distributed in the rectum (d).

**FIGURE 3:**
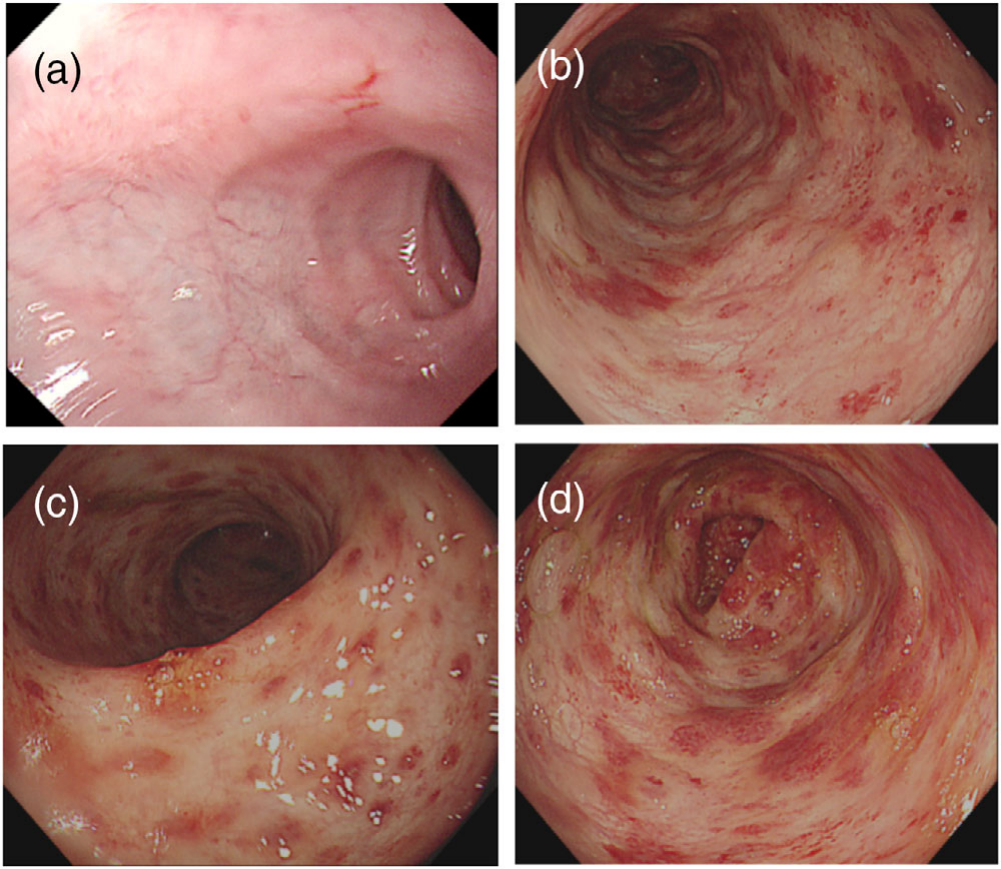
Endoscopic images of the patient at 19 months old. (a) Congestion and edema of the duodenal bulb mucosa. (b–d) Diffuse hyperemia, edema, erythema, erosion, and superficial ulcers observed from the distal ileum mucosa to the rectum.

**FIGURE 4:**
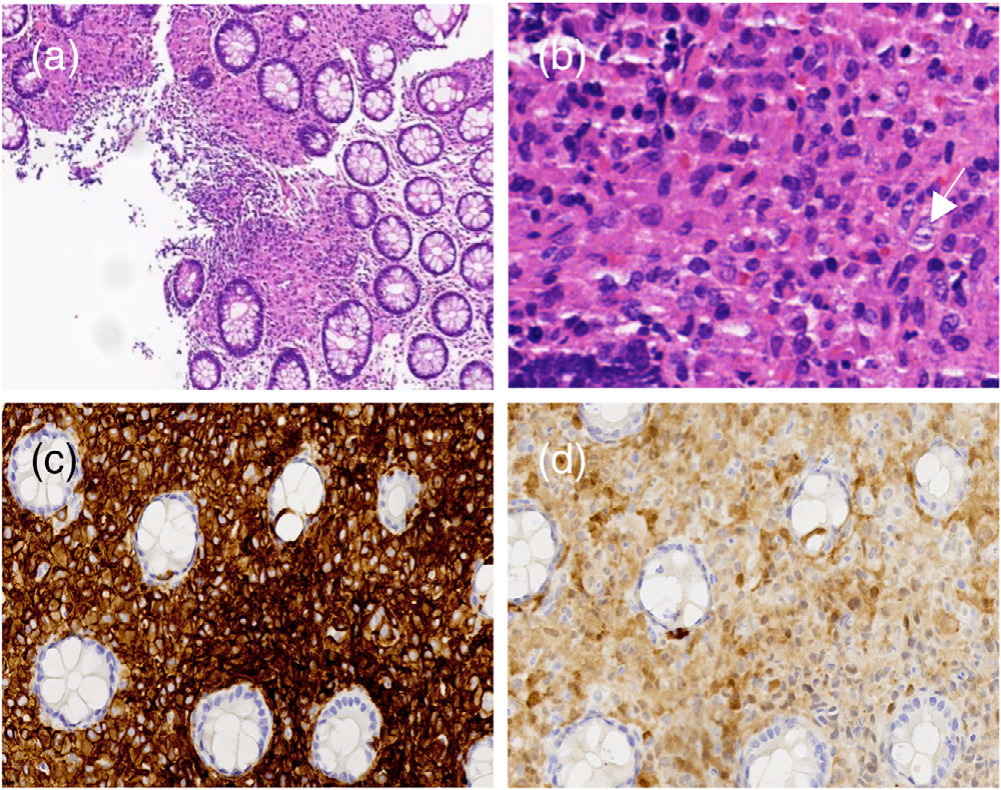
Hematoxylin and eosin staining showed that tumor cells were distributed in sheets and formed granuloma‐like structures (a, ×100); A typical Langerhans cell having eosinophilic cytoplasm, indistinct boundaries, vacuolated nuclei, and small nucleoli, was annotated by a white arrow (b, ×400). Immunohistochemical staining showed that CD1a was strongly positive (c, ×40), together with positive S‐100 (d, ×40).

At 1 year old, the patient underwent her first endoscopy, which revealed congestion, edema, and erythema of the duodenal bulb mucosa, along with chronic active inflammation in the rectal mucosa characterized by small vessel congestion and multifocal bleeding (Figure 1). Two months later, a second colonoscopy showed congested and edematous mucosa extending from the rectum to the ascending colon, with papular dense erythematous erosions and normal vascular patterns (Figure 2). Biopsies demonstrated congestion, edema, and chronic inflammatory cell infiltration with neutrophils and eosinophils (5‐15/HPF) in the colon and rectum, and some lymphocytic infiltration in the submucosa. Immunohistochemistry revealed CD68 positivity, negative acid‐fast bacilli, and positivity for Clostridium difficile. Based on her clinical history and colonoscopy findings, she was suspected to have inflammatory bowel disease, particularly Crohn's disease or eosinophilic gastroenteritis. Treatment with amino acid‐based formula, mesalazine, thalidomide, and oral prednisone led to a slight improvement in her diarrhea and bloody stools, but the symptoms persisted. Consequently, a third endoscopy was performed (Figure 3). While upper gastrointestinal endoscopy findings remained similar, the colorectal changes were markedly more severe compared to the initial findings. Erythema had progressed to diffuse hyperemia and edema with erythema erosion and superficial ulcers, extending even to the distal ileum mucosa. Typical Langerhans cells were found by tissue biopsy. Immunohistochemical staining was positive for CD1a, S‐100, CD68, and CD163, and genetic testing confirmed the presence of a BRAF p.V600E mutation, supporting the diagnosis of GIT‐LCH (Figure 4). Further investigations, including head imaging, bone scans, and bone marrow aspiration, were unremarkable. Whole exome sequencing identified a clinically unclear mutation in the COPA gene.

The patient demonstrated significant clinical improvement within a few days of initiating chemotherapy with vincristine and prednisone, combined with the BRAF inhibitor dabrafenib. In the first three months of treatment, the weight gain was relatively obvious. However, during chemotherapy in recent months, the weight gain was not obvious. At the 1‐year follow‐up, her quality of life had markedly improved, with normalization of bowel movements and a weight gain of 2.5 kg.

## DISCUSSION

We report a case with GIT‐LCH in a young girl who presented primarily with intractable diarrhea and bloody stools. She underwent three endoscopies but until the last time acquired a definitive diagnosis. The patient experienced symptom relief after treatment with chemotherapy and BRAF inhibitor. To our knowledge, this is the only reported GIT‐LCH case presenting with gastrointestinal symptoms and mild splenomegaly, in the absence of rash or osteolytic changes.

The clinical manifestations of GIT‐LCH are diverse and nonspecific, ranging from vomiting, abdominal pain, and persistent diarrhea to bloody stools, protein‐losing enteropathy, hypoproteinemia, and even intestinal perforation. It is commonly confused with infectious, allergic, immunodeficiency, and inflammatory bowel diseases. Our case was initially suspected to be Crohn's disease or very early onset inflammatory bowel disease. Notably, nearly all reported cases of GIT‐LCH present with a rash; however, our patient exhibited neither skin nor bone involvement, complicating the diagnostic process. This case underscores the importance of considering GIT‐LCH in infants presenting with intractable diarrhea and bloody stools, particularly in girls under the age of 2. A negative peripheral blood genetic test should not reduce clinical suspicion for LCH.

Endoscopy is vital for identifying the cause of chronic diarrhea and bloody stools. GI‐mucosa involvement in GIT‐LCH can extend from the esophagus to the rectum, with the duodenum and colon being the most frequently affected regions. The series of endoscopies in our case effectively illustrate the progression and evolution of GIT‐LCH. Notable colorectal changes included the progression from scattered erythema to diffuse hyperemia, erosion, and superficial ulcers, extending even to the distal ileal mucosa. Combining other case reports, multiple hemorrhagic spots, erosions, and ulcerations of the colorectal mucosa, as well as narrowing and erosion of the distal duodenum might be suggestive manifestations of GIT‐LCH on endoscopy.^3^, ^4^, ^5^ Given that Langerhans cell infiltration of the gastrointestinal wall may be continuous or discontinuous, we emphasize the importance of performing multipoint biopsies during colonoscopy. In our case, pathological examination of the first two biopsy specimens revealed chronic active mucosal inflammation, and until the third specimen, the characteristic coffee bean‐shaped nucleus of Langerhans cells was observed in the lamina propria.

Before LCH diagnosis, the patient was intermittently treated with prednisone due to suspected HPS and inflammatory bowel disease. The patient's diarrhea, with mucus and blood, slightly improved, although recurrently, after prednisone withdrawal. This phenomenon attracted our attention. Prednisone is a vital component of LCH chemotherapy drugs. Early prednisone treatment contributed to her long‐term relief, or it interfered with her clinical progress, so no rash appeared. However, Shima et al.^6^ reported the opposite results. An 11‐month‐old girl who suffered from continuous diarrhea, vomiting, and edema of the extremities was suspected to have allergic gastroenteropathy, but the administration of amino acid‐based milk produced no effects. She was administered prednisolone (2 mg/kg), which adversely affects immunodeficiency, and was subsequently complicated with life‐threatening cytomegalovirus‐associated HPS. These two very different views led us to consider whether early prednisolone use is effective or harmful, but further studies are needed for clarification.

Among patients with BRAF V600E‐mutated LCH, targeted therapy with BRAF inhibitors (dabrafenib) represents a novel therapeutic approach.^7^ Dabrafenib monotherapy alone or in combination with trametinib (a MEK inhibitor) has demonstrated clinical efficacy and manageable toxicity in patients with relapsed/refractory BRAF V600E–mutant pediatric LCH, with most responses ongoing.^8^, ^9^ The child was treated with chemotherapy and dabrafenib (2.5 mg/kg, twice per day) as first‐line therapy. Her symptoms of diarrhea and bloody stools disappeared quickly after 1 week, and her spleen gradually narrowed. At present, her treatment has lasted 1 year, her quality of life has significantly improved, she has normal bowel movements, and her weight has increased by 2.5 kg. This provides real‐world evidence for its pediatric application in GIT subsets. Prospective studies are warranted to further ascertain the long‐term efficacy and tolerability of dabrafenib, with or without MEK inhibition, as well as the duration of treatment.

In conclusion, we described a case with dynamic endoscopic findings and reflections on therapeutic drugs, aiming to enhance the understanding of GIT‐LCH. Early gastrointestinal endoscopy with multiple biopsies in suspected children can facilitate early detection. Dabrafenib is a promising treatment option for LCH. Further research is needed to clarify the relationship between GI manifestations, disease prognosis, and treatment outcomes.

## CONFLICT OF INTEREST STATEMENT

None.

## ETHICS STATEMENT

This study has been approved by the Human Research Ethics Committee of the Fourth Affiliated Hospital of Zhejiang University School of Medicine (Approval NO: K2024140).
